# Empagliflozin Use among Patients with Heart Failure in the Outpatient Department of Medicine in a Tertiary Care Centre

**DOI:** 10.31729/jnma.8288

**Published:** 2023-10-31

**Authors:** Rinku Ghimire, Sinet Pokharel, Raju Jayshwal, Rupesh Kumar Shreewastav

**Affiliations:** 1 Department of Pharmacology, Nobel Medical College Teaching Hospital (P) Ltd., Biratnagar, Morang, Nepal; 2 Department of Internal Medicine, Nobel Medical College Teaching Hospital (P) Ltd., Biratnagar, Morang, Nepal; 3 Department of Biochemistry, Nobel Medical College Teaching Hospital (P) Ltd., Biratnagar, Morang, Nepal

**Keywords:** *heart failure*, *outpatient care*, *patient safety*, *SGLT-2 inhibitors*

## Abstract

**Introduction:**

Sodium-glucose co-transporter 2 inhibitors were initially used in the treatment of type 2 diabetes mellitus and subsequently, it was shown to be useful in heart failure among patients with or without diabetes mellitus. This study aimed to find out the prevalence of empagliflozin use among patients with heart failure in an outpatient department of medicine in a Tertiary care centre.

**Methods:**

A descriptive cross-sectional study was conducted among patients with heart failure attending the outpatient Department of Internal Medicine from 1 December 2022 to 30 May 2023 after obtaining ethical approval from the Institutional Review Committee. All patients with heart failure in the given study period were included. A convenience sampling method was used. The point estimate was calculated at a 95% Confidence Interval.

**Results:**

Out of 550 patients, 188 (34.18%) (30.22-38.15, 95% Confidence Interval) received empagliflozin. The mean age was 55.11±9.99 years. A total of 124 (65.95%) were male. The mean duration of use was 104.97±63.16 days. Among the adverse effects, electrolyte imbalance was present in 14 (7.44%), hypotension in 14 (7.44%), and acute kidney injury in 12 (6.38%).

**Conclusions:**

The prevalence of empagliflozin use among patients with heart failure was found to be lower than mentioned in the guidelines.

## INTRODUCTION

Empagliflozin is a selective sodium glucose cotransporter 2 (SGLT-2) inhibitor used in the treatment of type 2 diabetes mellitus (T2DM). Subsequently, it was shown to have reduced cardiovascular events and nephropathy in patients with T2DM and established cardiovascular disease.^1,2^

Heart failure (HF) is a worldwide epidemic and one of the challenges faced by the healthcare system of all nations.^3^ Empagliflozin is a new class of drug that has been approved for the management of all patients with symptomatic HF.^4^ Empagliflozin is also the first among SGLT2 inhibitors to show beneficial effects in all HF subtypes.^4^ Data from clinical trials have shown that empagliflozin at doses of 10 mg and 25 mg is well tolerated.^5^

The objective of this study was to find out the prevalence of empagliflozin use among patients with HF in the outpatient department of medicine in a tertiary care centre.

## METHODS

This is a descriptive cross-sectional study among patients receiving Empagliflozin who attended the outpatient Department of Internal Medicine at Nobel Medical College Teaching Hospital (P) Ltd., Biratnagar, Morang, Nepal from 1 December 2022 to 30 May 2023.

Ethical approval was obtained from the Institutional Review Committee (Reference number: 724/2022). All patients with HF visiting the outpatient Department during the study period were included in the study. Patients with baseline hypotension, electrolyte imbalance and significant renal impairment (eGFR <30 ml/min) were excluded. A convenience sampling method was used. The sample size was calculated using the following formula:


$$\begin{array}{ll} \text{n} & ={\text{Z}}^{2}\times \frac{\text{p}\times \text{q}}{{\text{e}}^{\text{2}}}\\ & =1.96^{2}\times \frac{0.50\times 0.50}{0.05^{2}}\\ & =385 \end{array}$$

Where,

n = minimum required sample size
Z = 1.96 for a 95% Confidence Interval (CI)
p = prevalence taken as 50% for maximum sample size calculation
q = 1-p
e = margin of error, 5%

The minimum required sample size was 385. By adding a 10% non-response rate, the calculated sample size was 427. However, 550 patients were included. Age and gender distribution; comorbidities; duration of use of empagliflozin and adverse effects were recorded. Data was collected with the inclusion of pre-specified parameters in proforma.

Data were entered in Microsoft Excel 2007 and analysed using IBM SPSS Statistics version 20.0. The point estimate was calculated at a 95% CI.

## RESULTS

Among 550 patients, 188 (34.18%) (30.22-38.15, 95% CI) received empagliflozin. The mean age was 55.11±9.99 years with a predominance of males 124 (65.95%). Among co-morbidities, diabetes mellitus was present in 81 (43.08%), hypertension in 62 (32.97%), and coronary artery disease in 32 (17.02%) (Table 1).

**Table 1:** Baseline characteristics of patients receiving empagliflozin (n= 188).

Characteristics	n (%)
**Gender**	
Male	124 (65.95)
Female	64 (34.04)
**Comorbidities**	
Type 2 diabetes mellitus	81 (43.08)
Hypertension	62 (32.97)
Coronary artery disease	32 (17.02)
Dyslipidemia	29 (15.42)
Hypothyroidism	26 (13.82)
Renal impairment (eGFR <60 ml/min)	12 (6.38)

The mean heart rate was 55.11±9.99 beats per minute. and mean systolic and diastolic blood pressure was 121.66±19.94, and 75.34±11.92 mm Hg respectively (Table 2).

**Table 2:** Biochemical parameters (n = 188).

Parameters	Mean±SD
Haemoglobin (gm/dl)	12.05±1.60
Serum creatinine (mg/dl)	0.99±0.25
Mean serum sodium (meq/dl)	134.39±5.10
Mean serum potassium (meq/dl)	4.90±0.61
Random blood sugar (mg/dl)	141.64±37.57

Among the adverse effects, electrolyte imbalance was present in 14 (7.44%), out of which hyponatremia was present in 9 (4.78%) and hypo or hyperkalemia was present in 5 (2.65%). The drug was withdrawn due to intolerance in 9 (4.78%) for reasons like dizziness and not feeling well (Table 3).

**Table 3:** Adverse effects of empagliflozin use (n= 188).

Adverse effects	n (%)
Electrolytes imbalance	14 (7.44)
Hypotension	14 (7.44)
Acute kidney injury	12 (6.38)
Withdrawal due to other reasons	9 (4.78)
Urinary tract infection	1 (0.53)

Indications for empagliflozin were the presence of HF with diabetes mellitus in 107 (56.91%) (Table 3). The majority of patients 174 (92.55%) received 10 mg of empagliflozin per day (Table 3). The mean duration of empagliflozin use was 104.97±63.16 days (Table 4).

**Table 4:** Clinical indications and used dose of empagliflozin use (n= 188).

Characteristics	n (%)
**Indications for empagliflozin**	
Heart failure without diabetes mellitus	107 (56.91)
Heart failure with diabetes mellitus	81 (43.08)
**Dose of empagliflozin (per day)**	
10 mg	174 (92.55)
25 mg	14 (7.44)

## DISCUSSION

In this study, the prevalence of empagliflozin use was 34.18% among patients with HF which was found to be lower than mentioned in guidelines despite of beneficial effect on HF regardless of background HF therapies and irrespective of the presence or absence of T2DM.^4^

Empagliflozin is an oral drug that has been approved for use in the management of T2DM and HF. It has a good safety profile with a low incidence of adverse events.^6^ It works by reducing the reabsorption of glucose and increasing the excretion of glucose in urine. This leads to a reduction in blood glucose levels and has also been shown to have other beneficial effects on the cardiovascular system. Empagliflozin can be used as a single agent or as a combination with other antihyperglycemic drugs. It has also been approved for use to reduce the risk of cardiovascular death in patients with T2DM and heart failure.^3^ In addition, empagliflozin is effective in reducing body weight and blood pressure. The landmark trial (EMPA-REG OUTCOME), which pooled a total of 7020 patients found significantly lower rates of death from cardiovascular causes, hospitalizations for heart failure, and death from any cause.^7^ This trial also showed that empagliflozin did not increase the risk of major cardiac adverse events compared to placebo in patients with T2DM who were at high risk of cardiovascular events.^7^ In our study, this drug has been used in HF with or without T2DM though in a lesser number of patients.

Pooled data from different trials have shown that empagliflozin in both dose strengths 10 mg and 25 mg are well tolerated in patients with T2DM compared to placebo.^8^ High-daily doses had better efficacy and similar adverse effects as low doses in terms of HbA1c reduction and blood sugar control.^7^

The most common side effects reported in clinical trials were genital infections, UTIs, and thirst. However, these side effects were usually mild to moderate in severity and resolved with continued treatment. Empagliflozin increases urinary glucose concentration, thereby providing a favourable condition for pathogens to thrive leading to genital infections.^9^ Safety data pooled from randomized trials showed that patients on empagliflozin therapy had a higher incidence (3.6-4.2%) of genital infections as compared with patients on placebo therapy (0.7%).^10^ We had a case of UTI but did not find any patients reporting genital infections in our study. This could be due to underreporting owing to a milder form of genital infections.

However, no significant increase in the incidence of hypoglycemia and volume depletion has been noted in a study done in Bangladesh.^11^ We did not find any patients with hypoglycemia but hypotension and AKI were present in 6-7% of patients which could be due to concomitant use of other drugs like diuretics.

This is a hospital-based cross-sectional study representing patients from the eastern part of Nepal. Patients with HF were receiving other multiple drugs in combination and some of the adverse effects like electrolyte disturbances and hypotension attributed to empagliflozin could have been influenced by drugs like other diuretics and antihypertensive drugs.

## CONCLUSIONS

The prevalence of empagliflozin use among patients with HF was found to be lower than mentioned in the guidelines. Further, large studies are needed to determine the optimal use and safety profile of this drug in our patient population.
